# Comparison of the quality attributes of coconut waters by high‐pressure processing and high‐temperature short time during the refrigerated storage

**DOI:** 10.1002/fsn3.997

**Published:** 2019-03-25

**Authors:** Yan Ma, Lei Xu, Sujing Wang, Zhenzhen Xu, Xiaojun Liao, Yongyou Cheng

**Affiliations:** ^1^ Beijing Advanced Innovation Center for Food Nutrition and Human Health College of Food Science and Nutritional Engineering China Agricultural University Beijing Key Laboratory for Food Nonthermal Processing Key Lab of Fruit and Vegetable Processing Ministry of Agriculture Beijing China; ^2^ Institute of Quality Standard & Testing Technology for Agro‐Products Chinese Academy of Agricultural Sciences Key Laboratory of Agro‐food Safety and Quality Ministry of Agriculture Beijing China; ^3^ Institute of Agro‐products Storage and Processing Xinjiang Academy of Agricultural Sciences Urumqi China

**Keywords:** coconut water, high‐pressure processing, high‐temperature short time, quality

## Abstract

This study compared the shelf life and quality of high‐pressure processing (HPP) and high‐temperature short time (HTST)‐treated coconut water at 4°C. HPP of 500 MPa (5 min) and HTST of 72°C (15 s) treatments could ensure microbial safety of coconut water during refrigerated storage of 25 and 15 days, respectively. At the end of 15 days of storage, loss of 51.54% amino acids and 32.37% protein, and retention of 65.0% total sugars, 64.51% ascorbic acid, and 74.34% total phenols were found in HTST group. More nutrient contents, 76.85% amino acids, 76.76% total protein, and 93.17% total phenols, were retained in HPP groups at the end of 25 days of storage. HPP‐treated fresh‐like product could provide an effective approach of extending shelf life of coconut water.

## 1 INTRODUCTION

Coconut water, a clear liquid from coconut fruit, is regarded as a healthy drink as it is rich in calcium, magnesium, vitamin B, and vitamin C, which is one of the most popular beverages in tropical countries with unique flavor (Debmandal & Mandal, 2011). The water when taken out from the coconut spoils within a day because of contamination by microorganisms, which may be in the order of 10^6^ cfu/ml in the traditional way of collection (Balter et al., 2005). Even if the coconut water is extracted aseptically, air exposure still has negative effects on sensorial and nutritional qualities of the coconut water (Duarte, Coelho, & Leite, 2002).

Commercially available canned coconut water is given a high‐temperature/short‐time thermal treatment. Although the shelf life of thermally processed coconut water is long, its natural flavor and nutrient content were completely destroyed (Haseena, Kasturi Bai, & Padmanabhan, 2010). In recent years, there has been considerable interest in food preservation by nonthermal technologies, which minimize negative thermal effects on food nutritional and quality parameters (Knorr, 2003; Rawson et al., 2011; Tiwari, O'Donnell, & Cullen, 2009).

Nonthermal technologies of microfiltration (Junmee & Tongchitpakdee, 2015; Mahnot, Kalita, Mahanta, & Chaudhuri, 2014), high‐pressure carbon dioxide (HPCD) (Cappelletti et al., 2015), ultraviolet light C (Gautam et al., 2017), and ultrasound (Rojas, Trevilin, Funcia, Gut, & Augusto, 2017), have been applied to evaluate microbial degradation, enzymes inactivation, or extending shelf life of fresh coconut water. Concerted effect of microfiltration and l‐ascorbic acid addition proved to be a better method for processing coconut water than microfiltration alone (Das Purkayastha et al., 2012). The synergistic effect of supercritical carbon dioxide (SC‐CO_2_)+high power ultrasound was evident, and a higher microbial reduction was achieved compared to SC‐CO_2_ alone (Cappelletti, Ferrentino, & Spilimbergo, 2014). The inactivation kinetics of pressure‐assisted thermal processing (PATP) on the polyphenol oxidase (PPO) and peroxidase (POD) in coconut water followed the Weibull model, and color characteristics of which were well maintained (Chourio, Salais‐Fierro, Mehmood, Martinez‐Monteagudo, & Saldaña, 2018).

In this context, the goal of this study was to investigate the possibility of applying high pressure processing (HPP) to fresh coconut water in order to guarantee its microbial stability without addition of food additives, and monitored its comprehensive quality attributes during refrigerated storage. The high‐temperature short‐time processing (HTST) was used as a control group.

## 2 MATERIALS AND METHODS

### 2.1 Coconut and coconut water preparation

Mature coconuts (5.2 °Brix and pH 5.5) from Hainan province were purchased in a local supermarket (Beijing, China) and stored at 4°C for 24 hr prior to processing. These coconuts were opened, and coconut water was collected in three food grade tanks. For coconut water in the tanks 1 and 2, each 60 ml of natural coconut water was filled into polyethylene terephthalate bottles, and then the first group was immediately refrigerated at 4°C as the untreated group and the second groups were processed by HPP as described in Section 2.2. The coconut water in tank 3 was directly pasteurized by HTST equipment as described in Section 2.2.

### 2.2 Treatments

#### 2.2.1 High‐pressure processing

High‐pressure processing was accomplished in HPP‐650 (Baotou Kefa Co., Inner Mongolia, China). It has a stainless steel vessel (15 cm internal diameter × 30 cm internal height) with the pressure‐transmitting liquid of water inside. HPP‐650 pressurized at 120 MPa/min to reach 500 MPa, and the pressure‐release time was 10 s to depressurize to atmospheric pressure. This group of bottled coconut water was placed in the vessel and subjected to 500 MPa for 5 min, and this processing condition was selected based on our previous observation with modification (Xu, Lin, Wang, & Liao, 2015).

#### 2.2.2 High‐temperature short‐time processing

For HTST processing, the coconut water was pasteurized (72°C, 15 s), according to Regulation (EC) NO. 853/2004, in a pilot scale pasteurizer with a tubular heat exchanger (Armfield FT74, HTST/UHT Processing Unit, Hampshire, England). After pasteurization, the coconut water was aseptically filled into the identical polyethylene terephthalate bottles used in HPP after cooling to 20°C.

After processing, both groups were immediately refrigerated at 4°C.

### 2.3 Microbial analysis

As reported before, 20 ml of the coconut water was serially diluted with 0.85% sterile NaCl solution to 250 ml. Duplicated diluted samples (1.0 ml) were filled into the plates of appropriate agar. The plate count agar and the rose bengal agar were incubated at 36 ± 1°C (24 ± 2 hr) and at 28 ± 1°C (72 ± 2 hr) for detecting the viable cells of total aerobic bacteria (TAB) and molds and yeasts (M&Y), respectively (Xu et al., 2015).

### 2.4 Determination of total soluble solid, pH, and titratable acidity

Samples were measured at 25°C. Thermo Orion 868 pH meter (Thermo Fisher Scientific, Inc., MA, USA), WAY‐2S digital Abbe refraction meter (Shanghai Precision and Scientific Instrument Co., Shanghai, China), and 842 GPD titrino automatic potentiometric titrator (Metrohm, Switzerland) were used to measure the pH, total soluble solid (TSS), and titratable acidity (TA).

### 2.5 Color assessment

Color parameters of *L, a,* and *b* were measured with ColorQuest XE Colour Difference Meter from Hunter Associates Laboratory Inc. (Virginia, USA), illuminant D65, 10° Observer, in reflection mode. Total color difference (Δ*E*) was calculated using the equations provided in a previous study (Wang et al., 2014),

(1) $$\Delta E=\sqrt{{\left(L_{t}-L_{0}\right)}^{2}+{\left(a_{t}-a_{0}\right)}^{2}+{\left(b_{t}-b_{0}\right)}^{2}}$$

where *L_t_*,* a_t,_* and *b_t_* stand for the *L*,* a,* and *b* values, respectively, of the coconut water stored under 4°C at Days 1, 3, 6, 9, 15, 20, 25, and 30, and *L*_0_, *a*_0_, and *b*_0_ are values of the just‐prepared untreated coconut water.

### 2.6 Cloud and browning degree assessment

Ten milliliters of coconut water was centrifuged at 2,063 × *g*, 25°C for 10 min, and the absorbance of the supernatant at 660 nm was measured using a spectrophotometer (UV‐726 Shimadzu, Shanghai, China) for cloud assessment with a 1 cm path length cell (Cao et al., 2012).

Ten milliliters of coconut water was centrifuged at 5,157 × *g*, 6°C for 30 min, and then passed through cellulose nitrate membrane (0.45 μm), and the absorbance of the permeate at 420 nm was measured for browning degree (BD) using the spectrophotometer with a 1 cm path length cell (Cao et al., 2012).

### 2.7 Determination of total amino acids, total proteins, and total sugars

Total amino acids and total proteins assay kits were purchased from Nanjing Jiancheng Bioengineering Institute (Nanjing, China). Total amino acids and total proteins were determined using a Multiskan Go microplate spectrophotometer (Thermo Scientific, Waltham, USA) at the wavelength of 650 and 562 nm following the corresponding protocols, respectively. Total sugar content was determined by the anthrone method (Dreywood, 1946).

### 2.8 Determination of ascorbic acid, total phenols, and antioxidant capacity

Ascorbic acid test was carried out as described (Xu et al., 2015), coconut water (20 ml) was mixed with 2.5% metaphosphoric acid (100 ml), after incubation (4°C, 2 hr), the mixture was centrifuged at 5,157 × *g* (15 min, 4°C), and then the supernatant was removed and filtered through 0.45‐µm two‐layer cheese cloths for HPLC analysis. Total phenols were determined using the Folin–Ciocalteu method as described (Cao et al., 2012), and results were expressed as mg gallic acid/100 ml of the coconut water. Ferric‐reducing/antioxidant power (FRAP) was used to evaluate antioxidant capacity of samples.

### 2.9 Sensory evaluate

The procedure performed for sensory evaluation was described with modification (Wang et al., 2014). Twenty of graduate students from College of Food Science and Nutritional Engineering at China Agricultural University were trained to participate in the sensory tests. They were trained at least twice before sensory test. They were requested to mark the samples by their preference for aroma, flavor, color, and overall acceptability according to the score sheet standard shown in Table 1. The fresh coconut water and two groups of processed coconut water were served in randomly numbered scentless paper cups on a tray. A cup containing potable water and a piece of nonsalted cracker were also provided to them to eliminate the residual taste between samples.

**Table 1:** Standard score sheet for sensory evaluation of the coconut water

Scores	Color	Flavor	Mouthfeel	Overall acceptability
9 8 7	Transparent, no impurities	Appropriate proportion of coconut water flavor, pure aroma, no objectionable odor	Good mouthfeel. Appropriate consistency, refreshing, and exquisite	Excellent
6 5 4	Less transparent, a little amount of condensate	Generally appropriate proportion of coconut water flavor, pure a flavor, acceptable odor	General mouthfeel. Relatively consistency and refreshing	General
3 2 1	Turbid, anomalous color	No coconut water flavor, unacceptable off‐flavor	Bad mouthfeel. Inappropriate consistency, no refreshing	Unacceptable

### 2.10 Statistical analysis

Experiments were carried out in triplicate. Microorganisms and physicochemical characters were analyzed at Day 1, 3, 6, 9, 15, 20, 25, and 30 of storage and the other quality characters and sensory test were only carried for the samples with acceptable TAB and M&Y counts. All data were summarized by Microsoft Office 2013 Excel (Redmond, USA). An analysis of variance (ANOVA), curves fittings, and plotting drawings were finished using Origin 8.0 (OriginLab Corporation, Northampton, MA), and significance was established at *p* < 0.05.

## 3 RESULTS AND DISCUSSION

### 3.1 Effects of HPP and HTST on microorganisms and physicochemical characters

**Figure 1:**
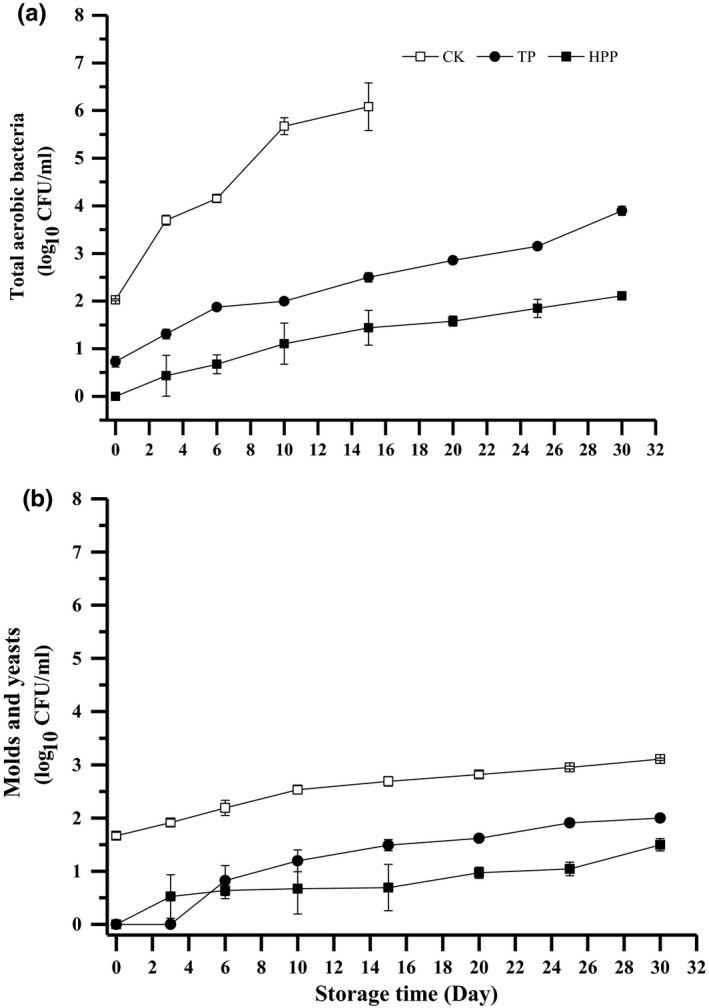
Total aerobic bacteria (TAB) and molds and yeasts (M&Y) of coconut water during storage (a, TAB; b, M&Y). HPP: high‐pressure processing

Initial counts of TAB and M&Y in the untreated coconut water are 2.03 ± 0.65 and 1.67 ± 0.85 log CFU/ml. According to the criteria mandated by National Food Safety Standard for Beverage (GB 7101‐2015), the acceptable TAB, molds, and yeasts in vegetable and fruit juice are less than 2, 1.3, and 1.3 log_10_CFU/ml, respectively. As shown in Figure 1, the counts of TAB and M&Y in HPP groups and M&Y in HTST group are undetectable, and the counts of TAB in HTST‐treated coconut water is 0.597 ± 0.02 log CFU/ml right after processing. The two treated groups show light microbial growth during the refrigerated storage comparing with control groups. The counts of HTST groups exceeds the acceptable limit on the 15th day of study, while the HPP indicates its effectiveness in ensure microbial safety during refrigerated storage of 25 days in this work.

As shown in Table 2, TSS, pH, and TA values show no significant difference after HPP and HTST treatment, and the values of them in HPP groups are relatively stable than HTST groups. The increase in TA was concomitant with the decrease in pH value in HTST groups, which could be due to the production of free acids by microbial growth (Das Purkayastha et al., 2012).

**Table 2:** Changes in pH, TSS and TA of coconut water treated during 25 days of storage at 4°C

Storage time (days)	Treatments	pH	TSS (°Brix)	TA (%)
0	Control	5.54 ± 0.01^a^	5.20 ± 0.17^ab^	0.075 ± 0.002^ab^
	HPP	5.56 ± 0.02^abc^	5.20 ± 0.20^a^	0.078 ± 0.001^abc^
	HTST	5.54 ± 0.008^a^	5.20 ± 0.10^ab^	0.075 ± 0.003^ab^
3	Control	5.55 ± 0.04^b^	5.30 ± 0.15^de^	0.081 ± 0.003^b^
	HPP	5.58 ± 0.01^abc^	5.20 ± 0.06^a^	0.076 ± 0.002^a^
	HTST	5.53 ± 0.03^a^	5.53 ± 0.12^c^	0.076 ± 0.002^ab^
6	Control	5.53 ± 0.02^c^	5.40 ± 0.12^e^	0.091 ± 0.003^c^
	HPP	5.66 ± 0.01^a^	5.20 ± 0.29^a^	0.079 ± 0.001^bc^
	HTST	5.57 ± 0.01^ab^	5.50 ± 0.00^c^	0.079 ± 0.001^bc^
9	Control	5.44 ± 0.02^d^	5.30 ± 0.01^cd^	0.087 ± 0.002^d^
	HPP	5.64 ± 0.01^bc^	5.10 ± 0.12^a^	0.078 ± 0.001^abc^
	HTST	5.56 ± 0.05^bc^	5.47 ± 0.15^c^	0.082 ± 0.002^cd^
15	Control	5.25 ± 0.03^e^	5.10 ± 0.00^bc^	0.095 ± 0.002^e^
	HPP	5.59 ± 0.02^abc^	5.10 ± 0.00^a^	0.080 ± 0.020^cd^
	HTST	5.57 ± 0.36^cd^	5.10 ± 0.00^a^	0.077 ± 0.001^ab^
20	Control	4.74 ± 0.43^f^	5.00 ± 0.17^b^	0.104 ± 0.001^f^
	HPP	5.59 ± 0.02^c^	5.00 ± 0.00^a^	0.078 ± 0.002^ab^
	HTST	5.28 ± 0.01^ab^	5.10 ± 0.06^a^	0.084 ± 0.001^d^
25	Control	5.10 ± 0.03^g^	4.70 ± 0.15^a^	0.112 ± 0.002^g^
	HPP	5.60 ± 0.01^ab^	5.00 ± 0.00^a^	0.082 ± 0002^d^
	HTST	5.20 ± 0.13^d^	5.30 ± 0.10^b^	0.089 ± 0.002^e^
30	Control	4.85 ± 0.34^h^	4.60 ± 0.00^a^	0.119 ± 0.002^h^
	HPP	5.61 ± 0.02^c^	5.00 ± 0.10^a^	0.080 ± 0.001^cd^
	HTST	5.28 ± 0.02^e^	5.20 ± 0.00^ab^	0.093 ± 0.002^f^

All data is mean ± *SD*, degrees of freedom=3.

Different superscripted letters represented a significant difference within the same column for each treatment (*p < 0.05*)

HPP: high‐pressure processing; TA: titratable acidity; TSS: total soluble solid.

### 3.2 Change in color parameters, cloud, and browning degree

As shown in Table 3, HPP slightly decreases the lightness (*L*) and raises the yellowness (*b*), while HTST shows more effect on redness (*a*). Similarly, a slight decrease in *L* values (from 99.59 to 98.35) and increase in *b* value (from 0.52 to 1.02) in HPCD‐treated coconut water, as well as higher *a* value after HTST compared to the control and HPP groups, were reported (Cappelletti et al., 2015). No pink color was observed in both HPP‐ and HTST‐treated coconut water during the storage, which might because of inactivation of PPO and POD here. The pink color in PATP‐treated coconut water was also not observed (Chourio et al., 2018). Δ*E* in the HPP‐treated coconut water ranges from 5.69 to 1.33 during the first 6 days of storage, while it is between 2.03 and 0.19 during the first 3 days, and quickly rises to 13.94 at the 6th day; for the final Δ*E*, HPP treatment of coconut water results in ∆*E* values ≤8 at the 25th day, while ∆*E* values >9.5 in HTST‐treated coconut water at the 15th day, separately (Table 3). Nevertheless, HPP groups showed more stable color attribute comparing with HTST groups. These color changes also agree with the cloudy appearance and browing degree of the coconut water in both treatments (Table 3). Considering that both treatments in this work were enough to control the color deterioration, we attributed the increasing cloudy and *L* values in HTST groups to the destabilization of emulsion and protein precipitation (Tangsuphoom & Coupland, 2005).

**Table 3:** Changes in colour parameters, cloudy and browning degree of coconut water during storage at 4°C

Storage time (days)	Treatments	*L*	*a*	*b*	*ΔE*	browning degree	cloudy
0	Control	77.40 ± 0.48	−1.78 ± 0.04	−3.13 ± 0.08	0.00	0.08 ± 0.01	94.20 ± 0.46
	HPP	72.25 ± 2.64	−1.62 ± 0.06	−1.73 ± 0.57	5.49	0.08 ± 0.01	94.87 ± 0.45
	HTST	75.49 ± 0.75	−2.09 ± 0.03	−3.69 ± 0.09	2.03	0.08 ± 0.01	91.87 ± 0.35
3	HPP	77.99 ± 1.29	−1.61 ± 0.13	−2.63 ± 0.43	0.79	0.08 ± 0.01	93.70 ± 0.46
	HTST	77.33 ± 1.54	−1.84 ± 0.31	−2.96 ± 1.14	0.19	0.13 ± 0.03	87.80 ± 0.56
6	HPP	78.63 ± 0.85	−1.90 ± 0.16	−2.63 ± 0.43	1.33	0.08 ± 0.01	94.36 ± 0.80
	HTST	91.25 ± 5.37	−2.62 ± 0.58	−4.55 ± 1.81	13.94	0.16 ± 0.03	83.87 ± 0.40
9	HPP	80.45 ± 1.30	−1.85 ± 0.18	−2.29 ± 0.86	3.16	0.09 ± 0.01	90.07 ± 0.67
	HTST	92.85 ± 1.17	−2.29 ± 0.36	−5.05 ± 0.88	15.58	0.27 ± 0.02	79.20 ± 0.87
15	HPP	70.15 ± 0.93	−1.65 ± 0.07	−2.43 ± 0.30	7.28	0.08 ± 0.01	88.46 ± 0.98
	HTST	86.85 ± 8.07	−2.43 ± 0.31	−5.53 ± 0.52	9.77	0.33 ± 0.03	73.20 ± 1.01
20	HPP	71.15 ± 0.33	−1.55 ± 0.11	–2.04 ± 0.54	6.34	0.09 ± 0.01	90.33 ± 1.10
	HTST	ND	ND	ND	ND	ND	ND
25	HPP	69.75 ± 1.47	−1.46+ ± 0.21	−2.33 ± 0.98	7.69	0.09 ± 0.01	88.03 ± 0.73
	HTST	ND	ND	ND	ND	ND	ND

All data were the Mean ± *SD*,* n* = 3.

HPP: high‐pressure processing.

### 3.3 Change in total amino acids, total proteins, and total sugars

The total amino acids, proteins, and sugars of untreated coconut water were 6.48 ± 0.32 g/L, 827.85 ± 20.47 mg/L, and 26.9 ± 0.46 g/L, respectively. Both HPP (500 MPa, 5 min) and HTST (72°C, 15 s) did not cause significant loss of total amino acids, proteins, and sugars.

**Figure 2:**
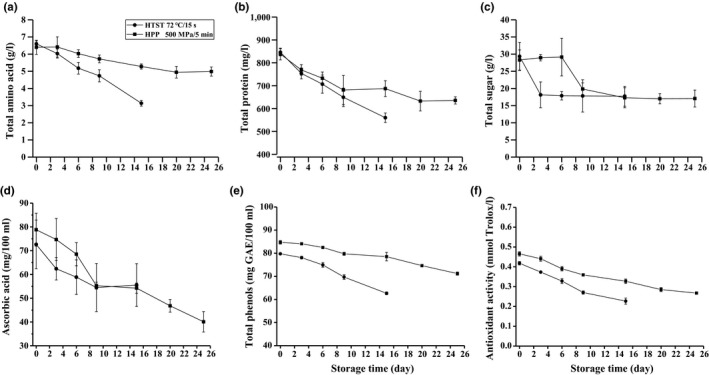
Total amino acids, total protein, total sugar, ascorbic acid, total phenols, and antioxidant capacity of coconut water during storage

As shown in Figure 2a,b, storage time has a significant effect (*p* < 0.05) on total amino acids and protein content in both groups; amino acids and protein loss are greater in HTST groups compared to HPP groups. At the 15th day, a loss of 51.54% amino acids and 32.37% protein was observed in HTST‐treated coconut water, while loss content of them was less in HPP‐treated ones, correspondingly 18.52% and 17.01%. At the 25th day, amino acids and protein contents of HPP‐treated ones were still higher than HTST‐treated coconut water at the 15th day, and only 23.15% amino acids and 23.24% protein were lost in the final products. Usually, protein decrease may be due to two reactions: (a) formation of complexes with other compounds like phenols forming phenoleprotein complex (Cheynier, 2005); (b) breakdown of proteins, which occurs normally in beverages during storage (Kulkarni & Aradhya, 2005). Degradation of proteins leads to the production of free amino acids, which are believed to be an end product of bacterial metabolism (Alexandrakis, Brunton, Downey, & Scannell, 2012). It was assumed that protein was degraded in this study, and an increase in amino acids should be synchronously found. Therefore, forming phenol–protein complex should be responsible for protein loss during storage here. Amino acids loss was attributed to reacting directly with the reducing sugars mainly, which is naturally present in the juice (Buedo, Elustondo, & Urbicain, 2000).

Unlikely, storage time shows a different influence on total sugars content in both HPP and HTST treated samples (Figure 2c), total sugars content decreases from day 0 to day 2, and remains stable for the 13 days in HTST groups, however, three stages are shown in HPP groups, in the initial stage of storage, it are stable from day 0 to day 6, later, it is reducing from day 6 to day 15, and finally, it is stable from day 15 to day 25 (percentage of surplus total sugars is almost 65%). Total sugars content was also found to decrease gradually in refrigerated and frozen bears seedless lime juices (Ziena, 2000). The increment of total sugars during storage was reported and was attributed to the breakdown of carbohydrates and starch (present mostly in immature fruits) into simple sugars (Das Purkayastha et al., 2012). The discrepancy in this study cloud is explained by different types and maturity level of coconuts. And, the decrease in total sugars during the storage for both groups may be attributed to either utilization of sugars by microbial action (Alexandrakis et al., 2012) or involvement of sugars in browning reactions (Das Purkayastha et al., 2012).

### 3.4 Change in ascorbic acid, total phenols, and antioxidant capacity

Ascorbic acid, total phenols, and antioxidant capacity of untreated coconut water were 86.09 ± 6.81 mg/100 ml, 84.28 ± 0.59 GAE mg/100 ml, and 0.52 ± 0.02 mmol Trolox/L, respectively. HPP did not cause significant loss of ascorbic acid, total phenols, and antioxidant capacity, while HTST resulted in a considerable reduction in them.

Ascorbic acid, total phenols, and antioxidant capacity in the HPP‐ and HTST‐treated coconut water during refrigerated storage are shown in Figure 2e–g, and remarkable decrease in ascorbic acid and total phenols is observed in both groups. At the 15th day, percentage of surplus ascorbic acid was 64.51% and 63.02% in HTST and HPP groups, and percentage of surplus total phenols was 74.34% and 93.17%, separately. At the 25th day, percentage of surplus ascorbic acid and total phenol in HPP‐treated coconut water was 46.57% and 84.46%. Loss of antioxidant capacity agreed with the loss of ascorbic acid and total phenols, and the antioxidant capacity in the HPP‐ and HTST‐processed coconut water using FRAP methods decreased with the increase in storage days, but more than 45% of antioxidant capacity was retained at the end of each storage period. Ascorbic acid loss was greater in HTST compared to HPP in the initial 6 days and then slowed down from day 9 to day 15 for both of them; it continued to reduce in HPP groups for the follow‐up 10 days. Ascorbic acid stability was dependent on the molar ratio of oxygen concentrations and ascorbic acid (Taoukis et al., 1998). Oxygen played a critical role in ascorbic acid stability at the atmospheric pressure, as well as the elevated pressure (Oey, Van der Plancken, Van Loey, & Hendrickx, 2008). The similar loss of ascorbic acid in the HTST‐ and HPP‐treated coconut water during day 9 to day 15 might be restricted by the limited oxygen content in the system. Lower ascorbic acid retention, comparing with total phenols, suggested that ascorbic acid might protect phenols from enzymatic degradation. Change in antioxidant capacity during refrigerated storage was agreed with previous studies in purple sweet potato nectar (Wang et al., 2012), strawberry juice (Cao et al., 2011), and mango nectar (Liu, Wang, Li, Bi, & Liao, 2014).

### 3.5 Sensory evaluation

**Figure 3:**
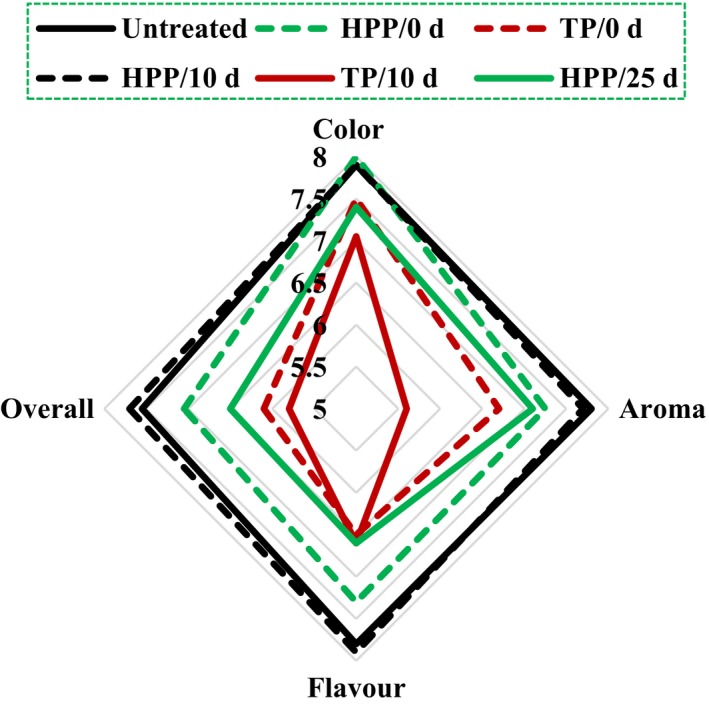
Sensory evaluation of coconut water

Sensory evaluations of HTST‐ and HPP‐processed coconut water, as well as untreated one, are shown in Figure 3. The untreated fresh coconut water achieved higher ratings in color, aroma, flavor, and overall acceptability. The color, aroma, flavor, and overall acceptability of the HPP‐treated coconut water were closer to that of the fresh coconut water. HTST group presented great color; however, its aroma, flavor, and overall acceptability achieved the lower ratings. A score of 5 was taken as the lower limit of acceptability here, and the overall score of the HTST‐treated coconut water was only 5.8 at day 10, while score of HPP‐treated one was 7.7 at day 10 and 6.5 at day 25. The sensory evaluations highlighted that HPP has less impact on the sensory attributes and maintained the original character of the coconut water than HTST. Similar positive results about sensory evaluation were also found in other HPP‐pasteurized navel orange juice (Baxter, Easton, Schneebeli, & Whitfield, 2005) and citrus juices (Hartyáni et al., 2011).

## 4 CONCLUSION

In conclusion, this study showed the applicability of HPP (500 MPa, 5 min) and HTST (72°C, 15 s) to fresh coconut water. The shelf life of the HTST‐treated coconut water samples was limited up to 15 days and that of HPP‐treated samples was extended to 25 days at 4°C. It is worth noting that utilization of HPP in coconut water substantially delayed losses of nutrient characters (such as total amino acids, proteins, sugar, ascorbic acid, phenols, and antioxidant capacity) as compared to HTST; HPP was superior to HTST in the intrinsic sensory quality assurance of coconut water, especially on original color and aroma. Currently, the commercial production of canned coconut water has employed a HTST preservation process and it eliminates the delicate flavor along with the microbes. From promoting product differentiation perspective, HPP‐treated fresh‐like coconut water could be a competitive option. There is no doubt that economic effectiveness of HPP should be considered as well, and microbiological shelf life stability and sensory properties of HPP‐treated coconut water should be further optimized in future product development.

## ETHICAL STATEMENT

This study does not involve any human or animal testing.

## CONFLICT OF INTEREST

The authors notify that there is no conflict of interest.

## AUTHOR CONTRIBUTIONS

Zhenzhen Xu interpreted the result, and drafted and reviewed the manuscript. Yan Ma collected and presented the data. Lei Xu processed the samples using HPP equipment. Sujing Wang and Yongyou Cheng involved in quality assessment experiments. Xiaojun Liao supervised the experiment.
